# Factors associated with return to work in young and middle-aged stroke survivors: a prospective cohort study with a focus on psychological resilience

**DOI:** 10.3389/fmed.2026.1780011

**Published:** 2026-03-18

**Authors:** Lili Wang, Xinping Bai, Xiaoxi Tan

**Affiliations:** Department of Neurology, Fuyang People’s Hospital, Fuyang, China

**Keywords:** influencing factors, logistic regression, psychological resilience, return to work, stroke

## Abstract

**Background:**

Return to work (RTW) is a core indicator for evaluating social functional recovery in young and middle-aged stroke survivors, holding significant importance for individuals, families, and society. However, RTW is influenced by multiple factors, and its predictive models remain underdeveloped, particularly regarding the independent contribution of psychosocial factors such as psychological resilience.

**Objective:**

To explore the multidimensional factors affecting RTW in young and middle-aged stroke survivors and to clarify the predictive value of psychological resilience.

**Methods:**

This prospective cohort study enrolled 253 young and middle-aged stroke survivors (aged 18–59 years) from Fuyang People’s Hospital (January 2022–December 2024). Baseline assessments included demographic characteristics, clinical severity [National Institutes of Health Stroke Scale (NIHSS), modified Rankin Scale (mRS)], psychological resilience [10-item Connor-Davidson Resilience Scale (CD-RISC-10)], and physiological indicators. Return to work (RTW) status was assessed at 6 months post-stroke. Multivariable logistic regression identified independent factors associated with RTW, with results reported as adjusted odds ratios (aOR) and 95% confidence intervals (CI). Model discrimination was evaluated using the area under the receiver operating characteristic curve (AUC).

**Results:**

At 6 months post-stroke, 131 participants (51.8%) had returned to work. In multivariable analysis, higher psychological resilience (aOR per 1-point increase: 1.149; 95% CI: 1.088–1.213; *p* < 0.001) and monthly income ≥8,000 CNY (aOR: 2.568; 95% CI: 1.376–4.793; *p* = 0.003) were independently associated with higher RTW likelihood, while older age (aOR per 1-year: 0.949; 95% CI: 0.917–0.982; *p* = 0.003) and higher NIHSS scores (aOR per 1-point: 0.818; 95% CI: 0.734–0.912; *p* < 0.001) were negatively associated. A comprehensive model incorporating clinical, socioeconomic, and psychological factors demonstrated excellent discrimination (AUC = 0.873; 95% CI: 0.830–0.916), significantly outperforming models containing only clinical (AUC = 0.781; *p* < 0.001) or clinical-socioeconomic (AUC = 0.826; *p* = 0.013) variables.

**Conclusion:**

The RTW status of young and middle-aged stroke survivors is jointly influenced by multiple factors, including age, severity of neurological deficits, economic income, and psychological resilience. Psychological resilience is a key protective predictive factor independent of traditional variables. Clinical rehabilitation assessment and intervention should integrate the evaluation of psychosocial resources, and implement early interventions for individuals with low psychological resilience to optimize their socio-occupational rehabilitation outcomes.

## 1 Introduction

The rising incidence of stroke, combined with advances in acute care, has resulted in increased survival rates, particularly among young and middle-aged adults. Consequently, return to work (RTW) has emerged as a critical indicator of social functional recovery and quality of life in this population (1). This demographic often bears significant familial and socioeconomic responsibilities. Failure to successfully resume work can lead to substantial loss of personal income, increased familial burden, and the onset of various psychosocial challenges (2). Therefore, identifying key factors that facilitate or hinder RTW is of immediate practical importance for developing targeted rehabilitation interventions and mitigating the socioeconomic burden of the disease.

Existing evidence indicates that RTW is a multidimensional and complex process, influenced by a wide array of factors encompassing demographic characteristics, the severity of post-stroke neurological deficits, and socioeconomic resources (3). For instance, survivors who are younger, have higher educational attainment, better financial status, and lower scores on the National Institutes of Health Stroke Scale (NIHSS) typically demonstrate a higher likelihood of RTW (4). In recent years, the application of the biopsychosocial model in chronic disease management has deepened, highlighting the significant role psychosocial factors play in disease prognosis (5).

Psychological resilience—defined as an individual’s capacity to maintain or rapidly regain positive adaptation when confronted with adversity, trauma, or significant stress (6)—has been recognized as an important psychological resource in chronic disease management. Within stroke rehabilitation, higher levels of psychological resilience have been associated with greater engagement in rehabilitation, more proactive coping strategies, and improved long-term functional outcomes (7). However, current research predominantly focuses on traditional clinical or sociological variables. Studies systematically evaluating psychological resilience as an independent, core predictor and assessing its incremental predictive value relative to other factors remain insufficient (8). Much of the existing literature employs cross-sectional designs, limiting causal inference, or suffers from limited sample sizes that fail to comprehensively control for various confounding factors (9). Furthermore, the mechanisms through which psychological resilience interacts with neurological status and socioeconomic conditions to jointly influence RTW decisions and processes require further elucidation (10).

Despite growing recognition of psychological factors in stroke recovery, several gaps remain in the literature on RTW prediction. First, most predictive models for post-stroke RTW have focused predominantly on clinical severity measures (e.g., NIHSS, mRS) and basic demographic characteristics, with limited integration of standardized assessments of psychological constructs (11). Second, among the few studies that have examined psychological resilience, it has typically been analyzed as a secondary or mediating variable, and its independent predictive contribution beyond traditional clinical and socioeconomic factors remains inadequately quantified (12). Third, evidence from Western populations may not directly generalize to Chinese cultural contexts, where family support structures, workplace norms, and conceptualizations of resilience may differ (13). Methodologically, many existing studies have employed cross-sectional designs or have not adequately controlled for potential confounders, limiting the robustness of their conclusions (14). Consequently, there is a need for prospective studies that systematically evaluate whether psychological resilience provides incremental predictive value for RTW when added to established clinical and socioeconomic variables, particularly in non-Western healthcare settings (15).

To address these gaps, this study aims to prospectively and systematically investigate the predictive value of psychological resilience for RTW in young and middle-aged stroke survivors. We incorporated the 10-Item Connor-Davidson Resilience Scale (CD-RISC-10), a reliable and valid instrument, into the assessment battery. The primary objective is to determine whether psychological resilience retains an independent predictive effect on RTW after controlling for traditional clinical severity and socioeconomic factors. By constructing and comparing predictive models with different dimensional combinations, this study seeks to reveal the incremental contribution of psychological resilience in optimizing RTW prediction. The findings are expected to provide empirical evidence for developing a more comprehensive post-stroke rehabilitation assessment framework and to indicate directions for creating individualized vocational rehabilitation programs incorporating psychological skill training.

## 2 Materials and methods

### 2.1 Study participants and design

This was a single-center, prospective observational cohort study. Participants were consecutively recruited from young and middle-aged stroke survivors admitted to the Department of Neurology, Fuyang People’s Hospital between January 2022 and December 2024, who met the predefined inclusion criteria. The study involved no active intervention, aiming to observe disease outcomes and related influencing factors under natural conditions.

Sample size calculation was based on the primary outcome: RTW status (a dichotomous variable) at 6 months post-stroke. This calculation is a fundamental component of rigorous clinical research design, ensuring sufficient statistical power to detect clinically meaningful effects and avoiding false-negative results or resource waste due to an underpowered study. Estimation was performed using G*Power software (version 3.1.9.7). Parameters were set as follows: test type = “binary logistic regression” (the primary analysis model); effect size measure = odds ratio (OR). Based on preliminary pilot data, the expected 6-month RTW rate in the control group (low resilience level) was approximately 45%. A clinically meaningful effect was defined as an OR of 1.88, from which corresponding probability values were calculated. The alpha error probability (two-tailed) was set at 0.05, and the statistical power (1-*β*) was set at 0.80 (i.e., *β* = 0.2). Under these parameters, the calculated minimum required sample size was 220 participants. Considering the potential for loss to follow-up common in prospective cohort studies, and referencing similar studies, an attrition rate of approximately 15% was anticipated. Accordingly, the final planned recruitment target was *N* = 220/(1–0.15) ≈ 260 participants. By the end of the final follow-up in June 2025, complete data were available for 253 participants, while seven participants were lost to follow-up (attrition rate 2.7%). The final analysis was conducted on the 253 participants with complete data.

### 2.2 Inclusion and exclusion criteria

Clear inclusion and exclusion criteria were established to construct a homogeneous cohort capable of effectively addressing the research question.

#### Inclusion criteria

(1) Aged between 18 and 59 years, defined as the “young and middle-aged” working population.

(2) Diagnosed with ischemic or hemorrhagic stroke, either first-ever or recurrent, confirmed by cranial computed tomography (CT) or magnetic resonance imaging (MRI) and meeting the World Health Organization (WHO) diagnostic criteria for stroke.

(3) The index stroke event occurred on or after January 1, 2022.

(4) Had paid employment prior to the stroke onset. This included formal full-time or part-time positions, as well as informal work such as hourly, temporary, or gig economy jobs. Employment status was verified through available documentation, including social security contribution records, bank statements showing regular income, employment contracts, or, for informal workers, a self-reported work history corroborated by a family member. A minimum average of 20 working hours per week over the 3 months preceding the stroke was required to ensure a defined pre-stroke work role. This criterion aimed to ensure participants had a defined work role and the potential for RTW.

(5) Provided informed consent voluntarily signed by the patient or their legal proxy.

#### Exclusion criteria

(1) Presence of significant cognitive impairment, defined as a score below 24 on the Mini-Mental State Examination (MMSE), to ensure the ability to comprehend and complete the questionnaire assessments in this study.

(2) Comorbid with advanced malignant tumors, end-stage renal disease (requiring regular dialysis), decompensated liver cirrhosis, or other major diseases with an expected lifespan of less than 2 years or those likely to severely impact functional prognosis.

(3) Presence of severe communication barriers, such as global aphasia or severe sensory aphasia, assessed by researchers as preventing effective cooperation to complete follow-up assessments.

(4) Pregnant or lactating women.

(5) Planned to relocate outside the study region during the follow-up period or explicitly stated inability to cooperate with at least one follow-up assessment.

### 2.3 Research methods

This study strictly adhered to observational cohort study reporting standards and implemented multiple quality control measures to enhance data reliability and research reproducibility.

(1) Study design and follow-up procedure: A prospective cohort study was conducted with strict attention to temporal sequencing. The baseline assessment (T0) was performed at the time of clinical stabilization prior to hospital discharge (median 12 days post-stroke; IQR: 8–18 days). For the minority of participants (*n* = 42, 16.6%) recruited during outpatient follow-up (due to missed inpatient enrollment), baseline assessment was completed within 4 weeks post-discharge (median 24 days post-stroke; IQR: 21–30 days). Critically, for all participants, we verified at the time of baseline assessment that they had not yet returned to work. Participants who had already resumed any paid work prior to baseline assessment were excluded (*n* = 3) to ensure temporal precedence of all predictor variables. Subsequently, systematic follow-ups were conducted at 3 months (interim contact) and 6 months post-stroke (primary endpoint) via standardized structured interviews (primarily during outpatient visits, supplemented by telephone interviews). This design ensured that all predictor variables were measured before the outcome (RTW) could occur, thereby minimizing the risk of reverse causation bias.

(2) Data collection procedures and personnel training: All data collection was performed by three research nurses who underwent unified, rigorous training. Training covered: study protocol interpretation, standard scoring rules for various scales, standardized procedures for measuring blood pressure and blood glucose, and patient communication techniques. For clinical measures (e.g., NIHSS, mRS) and laboratory data, a pre-designed standardized Case Report Form (CRF) was used for recording. Psychological scales (CD-RISC-10) were self-administered by patients in a quiet environment. For patients with limb mobility impairments that did not affect reading, researchers read each item aloud, the patient indicated their choice, and the researcher marked the response, ensuring a standardized assessment process.

(3) Core definition and assessment of return to work status: The operational definition of ‘Return to Work’ was explicitly defined following established guidelines (6). ‘Return to Work’ was defined as resuming any form of paid work at 6 months post-stroke, including both formal employment (full-time or part-time positions with employment contracts) and informal work (e.g., hourly, temporary, gig economy, or self-employed work). The definition required accumulating an average of ≥20 h of work per week, sustained for at least 4 consecutive weeks.

*Verification*: RTW status was verified through a combination of methods: (a) participant self-report using a structured work status questionnaire; (b) confirmation by a family member when available; and (c) for participants in formal employment, verification through employment records or social security contribution statements when participants provided consent for such verification (*n* = 186, 73.5%). For participants in informal work, verification relied on self-report corroborated by family members, as formal employment records were not available.

*Pre-stroke employment status*: To be included, participants were required to have had paid employment prior to the stroke. Individuals who were actively seeking employment but unemployed at the time of stroke were not eligible for inclusion, as the concept of ‘returning’ to a job would not apply. This criterion was strictly enforced to ensure a homogeneous sample with a defined pre-stroke work role.

(4) Comprehensive quality control system:

*Data management*: A double-data entry mode was adopted, followed by cross-verification and logical error checking to ensure data transcription accuracy.

*Measurement standardization*: All equipment for measuring physiological indicators underwent regular metrological calibration. Blood pressure monitors were calibrated monthly using a standard mercury sphygmomanometer for parallel comparison; blood glucose meters were verified with manufacturer-provided control solution before each new batch of test strips.

*Ethical compliance*: The study protocol and informed consent forms were reviewed and approved by the Ethics Committee of Fuyang People’s Hospital (Approval No.: 2022–159; Date of approval: January 13, 2022). This was a full-board review approval (Category: Expedited Review for observational studies involving survey procedures and standard clinical assessments). The study was conducted in strict accordance with the principles of the Declaration of Helsinki. Written informed consent was obtained from all participants or their legal proxies prior to enrollment.

### 2.4 Observed indicators

(1) Primary outcome measure: *return to work status*. As described above, assessed via structured interview at 6 months post-stroke and recorded as a binary variable (yes = 1, no = 0). This definition focuses on economically meaningful work participation, a key endpoint in rehabilitation medicine and socio-economic research.

(2) Core predictor: *psychological resilience*. Measured using the Chinese version of the 10-item Connor-Davidson Resilience Scale (CD-RISC-10). This scale comprises 10 items, each rated on a 5-point Likert scale from 0 (“Not true at all”) to 4 (“True nearly all the time”), yielding a total score range of 0–40. Higher scores indicate a greater ability to maintain adaptation and recovery when facing stress, adversity, or trauma. This Chinese version has demonstrated good reliability and validity in Chinese populations. The Cronbach’s alpha coefficient was 0.895 in this study. Assessed at baseline (T0).

(3) Neurological deficit severity. Assessed using the National Institutes of Health Stroke Scale (NIHSS). This scale includes 15 items evaluating level of consciousness, gaze, visual fields, facial palsy, motor function, ataxia, sensation, language, dysarthria, and neglect. Total scores range from 0 to 42, with higher scores indicating more severe neurological deficits. Assessments were performed by certified neurologists at hospital admission and at baseline assessment (T0).

(4) Disability and functional independence level. Assessed using the Modified Rankin Scale (mRS). This is the most commonly used functional outcome measure in global stroke clinical trials, gauging disability levels post-stroke. Scores range from 0 (no symptoms) to 5 (severe disability, bedridden, requiring constant care), with 6 indicating death. An mRS score ≤2 is typically defined as functional independence or mild disability, while ≥3 indicates moderate-to-severe disability or functional dependence. Assessed at baseline (T0) and each follow-up time point (9).

(5) Blood pressure levels. As a basic vascular risk indicator, measured using an internationally standardized (ESH, AAMI) validated upper-arm electronic blood pressure monitor (Omron HEM-7124). Patients were instructed to rest seated for 5 min before measurement. Blood pressure was measured in the right upper arm. Three consecutive measurements were taken at least 1 min apart, and the average of the last two readings was recorded as the final systolic and diastolic blood pressure (unit: mmHg).

(6) Glycemic status indicators. Included fasting blood glucose and glycated hemoglobin (HbA1c). Fasting blood glucose was measured using capillary blood from a fingertip sample after at least 8 h of fasting, using the same brand of glucose meter and test strips (Roche Accu-Chek Performa) (unit: mmol/L). HbA1c, reflecting average blood glucose levels over the past 2–3 months, was measured from venous blood samples sent to the central laboratory and analyzed using high-performance liquid chromatography (HPLC) (unit: %).

(7) Lipid profile. As important markers of atherosclerotic risk, the following indicators were measured from fasting venous blood samples using an automated biochemical analyzer: Total Cholesterol (TC), Triglycerides (TG), High-Density Lipoprotein Cholesterol (HDL-C), and Low-Density Lipoprotein Cholesterol (LDL-C) (units: mmol/L). LDL-C was typically calculated using the Friedewald formula (when TG < 4.5 mmol/L) or measured directly.

(8) Plasma homocysteine. Hyperhomocysteinemia is an independent risk factor for cardiovascular and cerebrovascular diseases. Fasting venous blood was collected, and quantitative analysis was performed using an enzymatic cycling method on a biochemical analyzer (unit: μmol/L). The normal upper reference limit is typically 15 μmol/L (10).

(9) Secondary outcomes: Data on partial RTW (e.g., reduced hours, modified duties) or job change were not systematically collected in this study; we acknowledge this as a limitation and have noted it in the Discussion section.

### 2.5 Statistical methods

Data analysis was performed using SPSS statistical software (version 26.0; IBM Corp.) and R language (version 4.2.0). All statistical tests were two-tailed, and a *p*-value <0.05 was considered statistically significant.

First, descriptive statistics were computed for the entire sample. Continuous variables conforming to a normal distribution were presented as mean ± standard deviation (mean ± SD); non-normally distributed variables were presented as median and interquartile range [median (IQR)]; categorical variables were described using frequencies and percentages (*n*, %).

Second, univariate analyzes were conducted for preliminary variable screening. Differences in baseline indicators between the RTW group and the non-RTW group were compared. For continuous variables, independent samples *t*-test was used for normally distributed data with homogeneity of variance; otherwise, the Mann–Whitney *U* test was employed. For categorical variables, the chi-square test (*χ*^2^ test) or Fisher’s exact test (when more than 25% of cells had an expected count <5) was used.

Finally, multivariate analysis models were constructed to identify independent factors influencing RTW and to quantify the effect size of the core predictor—resilience. Using RTW status at 6 months post-stroke (yes = 1) as the dependent variable, variables with *p* < 0.10 in univariate analysis and those deemed important based on clinical knowledge (e.g., age, sex, stroke type) were included as candidate independent variables in a binary logistic regression model. Variable selection was performed using the Forward Likelihood Ratio (Forward LR) method to control for confounding effects. Results are presented as adjusted Odds Ratios (ORs) with their 95% Confidence Intervals (CIs). Model goodness-of-fit was evaluated using the Hosmer-Lemeshow test, where *p* > 0.05 indicates a good fit. The discriminative ability of predictive models was assessed using the area under the receiver operating characteristic curve (AUC). Comparisons between AUCs of different models were performed using the non-parametric DeLong test for correlated ROC curves. We report AUC values with their 95% confidence intervals. It is important to note that these AUCs reflect in-sample (apparent) performance and may be optimistic due to overfitting. Internal validation using bootstrapping (200 repetitions) yielded optimism-corrected AUC estimates within 0.02 of the apparent values, suggesting minimal overfitting. Nevertheless, external validation in independent cohorts is necessary before clinical application.

## 3 Results

### 3.1 Comparison of baseline characteristics of study participants

Table 1 compares the baseline characteristics between the Return to Work group (RTW group) and the Non-Return to Work group (non-RTW group). The RTW group demonstrated significantly more favorable outcomes compared to the non-RTW group regarding age, years of education, proportion with a monthly income ≥8,000 CNY, NIHSS score, proportion with mRS ≤ 2, CD-RISC-10 total score, fasting blood glucose, glycated hemoglobin (HbA1c), LDL-C, and homocysteine levels. The corresponding statistical values were *t* = −4.503, *t* = 6.372, *χ*^2^ = 21.093, *Z* = -7.254, *χ*^2^ = 48.527, *t* = 8.528, *t* = −4.047, *t* = −4.542, *t* = −3.294, *t* = −5.080, respectively, with all *p*-values <0.05. No statistically significant differences were observed for other variables such as sex, stroke type, or history of hypertension (see Table 1).

**Table 1:** Comparison of baseline characteristics between the return to work and non-return to work groups.

Characteristic	Total sample (*n* = 253)	RTW group (*n* = 131)	Non-RTW group (*n* = 122)	Statistical value	*p*-value
Demographic characteristics					
Age (years), mean ± SD	52.34 ± 8.67	50.12 ± 8.23	54.71 ± 8.41	*t* = −4.503	<0.001
Male, *n* (%)	158 (62.5)	84 (64.1)	74 (60.7)	*χ*^2^ = 0.331	0.565
Years of education, mean ± SD	11.27 ± 3.85	12.63 ± 3.52	9.82 ± 3.61	*t* = 6.372	<0.001
Pre-stroke monthly income ≥8,000 CNY, *n* (%)	142 (56.1)	91 (69.5)	51 (41.8)	χ^2^ = 21.093	<0.001
Clinical characteristics					
Stroke type (ischemic), *n* (%)	218 (86.2)	115 (87.8)	103 (84.4)	*χ*^2^ = 0.620	0.431
Baseline NIHSS score, median (IQR)	5 (3, 8)	4 (2, 6)	7 (5, 10)	*Z* = −7.254	<0.001
mRS ≤ 2 at baseline, *n* (%)	167 (66.0)	112 (85.5)	55 (45.1)	*χ*^2^ = 48.527	<0.001
History of hypertension, *n* (%)	162 (64.0)	81 (61.8)	81 (66.4)	*χ*^2^ = 0.611	0.434
History of diabetes, *n* (%)	78 (30.8)	36 (27.5)	42 (34.4)	*χ*^2^ = 1.434	0.231
History of smoking, *n* (%)	109 (43.1)	59 (45.0)	50 (41.0)	*χ*^2^ = 0.449	0.503
History of alcohol use, *n* (%)	95 (37.5)	52 (39.7)	43 (35.2)	*χ*^2^ = 0.557	0.455
Functional and psychological scores					
CD-RISC-10 total score, mean ± SD	30.73 ± 5.68	33.27 ± 4.12	28.05 ± 5.83	*t* = 8.528	<0.001
Physiological and biochemical indicators					
Systolic BP (mmHg), mean ± SD	138.42 ± 16.73	136.89 ± 15.24	140.05 ± 18.07	*t* = −1.569	0.118
Diastolic BP (mmHg), mean ± SD	84.56 ± 11.38	83.72 ± 10.91	85.44 ± 11.83	*t* = −1.238	0.217
Fasting blood glucose (mmol/L), mean ± SD	6.28 ± 1.97	5.83 ± 1.62	6.76 ± 2.21	*t* = −4.047	<0.001
HbA1c (%), mean ± SD	6.51 ± 1.32	6.18 ± 1.04	6.87 ± 1.49	*t* = −4.542	<0.001
Total cholesterol (mmol/L), mean ± SD	4.65 ± 1.07	4.59 ± 1.02	4.71 ± 1.12	*t* = −0.936	0.35
Triglycerides (mmol/L), median (IQR)	1.52 (1.11, 2.03)	1.48 (1.09, 1.98)	1.57 (1.16, 2.11)	*Z* = −1.432	0.152
HDL-C (mmol/L), mean ± SD	1.21 ± 0.31	1.23 ± 0.29	1.18 ± 0.33	*t* = 1.335	0.183
LDL-C (mmol/L), mean ± SD	2.78 ± 0.86	2.62 ± 0.79	2.95 ± 0.89	*t* = −3.294	0.001
Homocysteine (μmol/L), mean ± SD	14.86 ± 5.17	13.41 ± 4.23	16.42 ± 5.61	*t* = −5.080	<0.001

### 3.2 Univariate analysis of factors associated with return to work status

Table 2 presents the results of the univariate logistic regression analysis, with Return to Work status (RTW vs. Non-RTW) as the dependent variable. Independent variables included continuous variables (e.g., age, years of education; assigned per 1-unit increase) and categorical variables (e.g., male vs. female, monthly income ≥8,000 CNY vs. <8,000 CNY). The Wald *χ*^2^ values for age, years of education, monthly income, NIHSS score, mRS, CD-RISC-10 total score, fasting blood glucose, HbA1c, LDL-C, and homocysteine were 19.237, 39.011, 20.693, 49.122, 46.827, 65.674, 16.187, 19.876, 10.676, and 24.735, respectively, with *p* < 0.05, indicating significant OR values. Sex and stroke type showed no significant associations (see Table 2).

**Table 2:** Univariate analysis of factors influencing return to work in young and middle-aged stroke survivors.

Variable	*β*-value	Std. error	Wald *χ*^2^ value	*p*-value	Crude OR	95% CI
Age (per 1-year increase)	−0.058	0.013	19.237	<0.001	0.944	0.919–0.969
Male (vs. female)	0.144	0.25	0.334	0.563	1.155	0.709–1.883
Years of education (per 1-year increase)	0.202	0.032	39.011	<0.001	1.224	1.149–1.304
Monthly income ≥8,000 CNY (vs. <8,000)	1.163	0.256	20.693	<0.001	3.201	1.939–5.285
Ischemic stroke (vs. hemorrhagic)	0.263	0.335	0.619	0.431	1.301	0.675–2.506
NIHSS score (per 1-point increase)	−0.265	0.038	49.122	<0.001	0.767	0.713–0.826
mRS ≤ 2 (vs. >2)	1.815	0.265	46.985	<0.001	6.145	3.656–10.328
CD-RISC-10 total score (per 1-point increase)	0.184	0.023	65.674	<0.001	1.202	1.149–1.258
Fasting blood glucose (per 1 mmol/L increase)	−0.241	0.06	16.187	<0.001	0.786	0.699–0.884
HbA1c (per 1% increase)	−0.398	0.089	19.876	<0.001	0.672	0.564–0.800
LDL-C (per 1 mmol/L increase)	−0.425	0.13	10.676	0.001	0.654	0.507–0.843
Homocysteine (per 1 μmol/L increase)	−0.106	0.021	24.735	<0.001	0.9	0.863–0.938

### 3.3 Multivariate logistic regression analysis of independent factors influencing return to work

**Figure 1:**
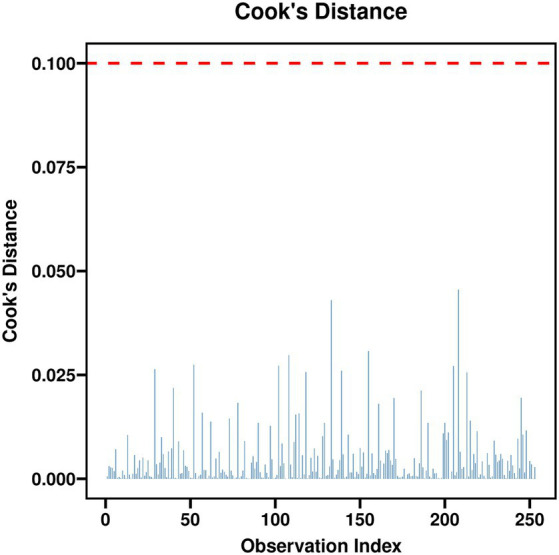
Cook’s distance plot.

**Figure 2:**
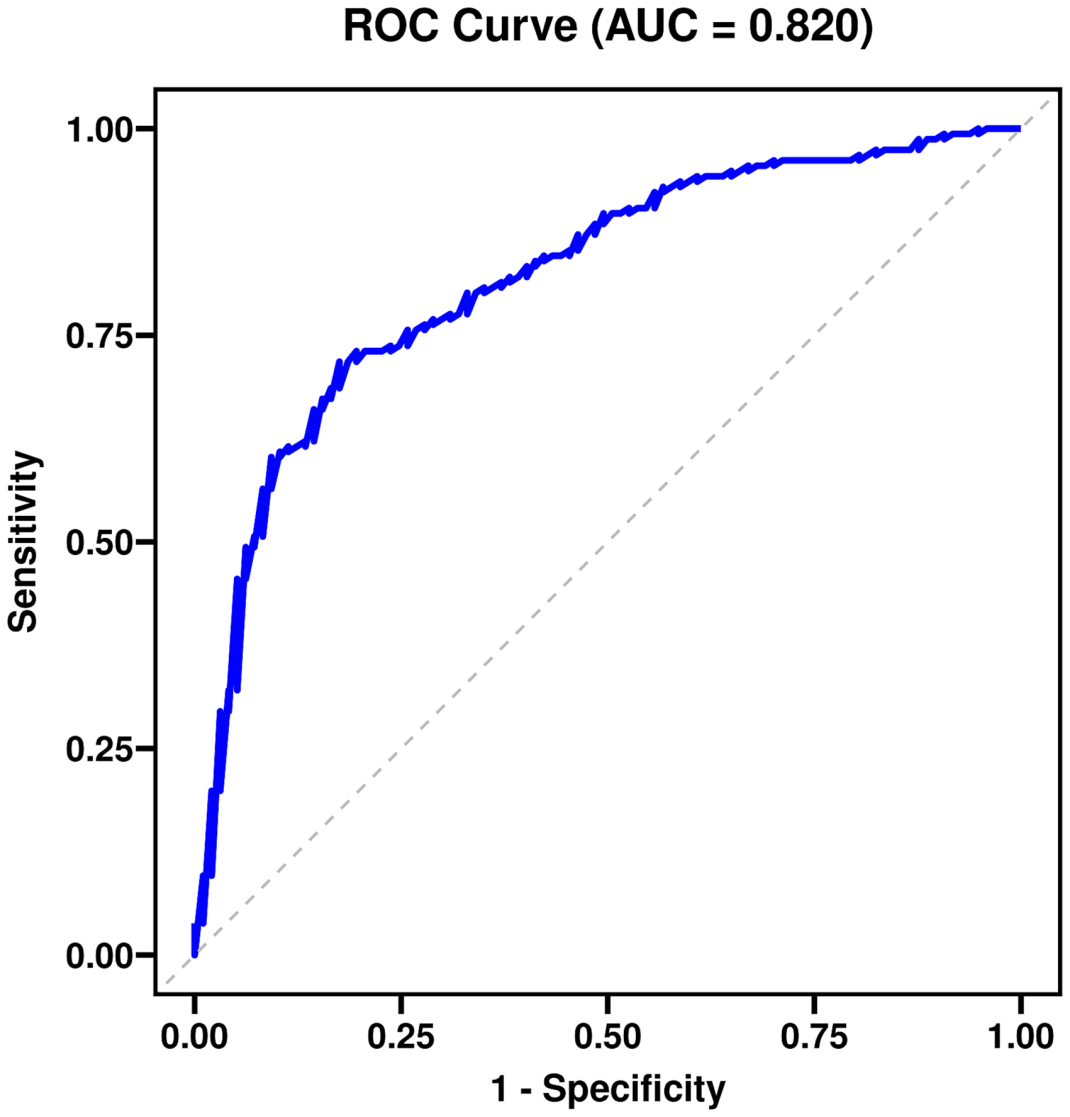
Receiver operating characteristic (ROC) curve for the predictive model.

**Figure 3:**
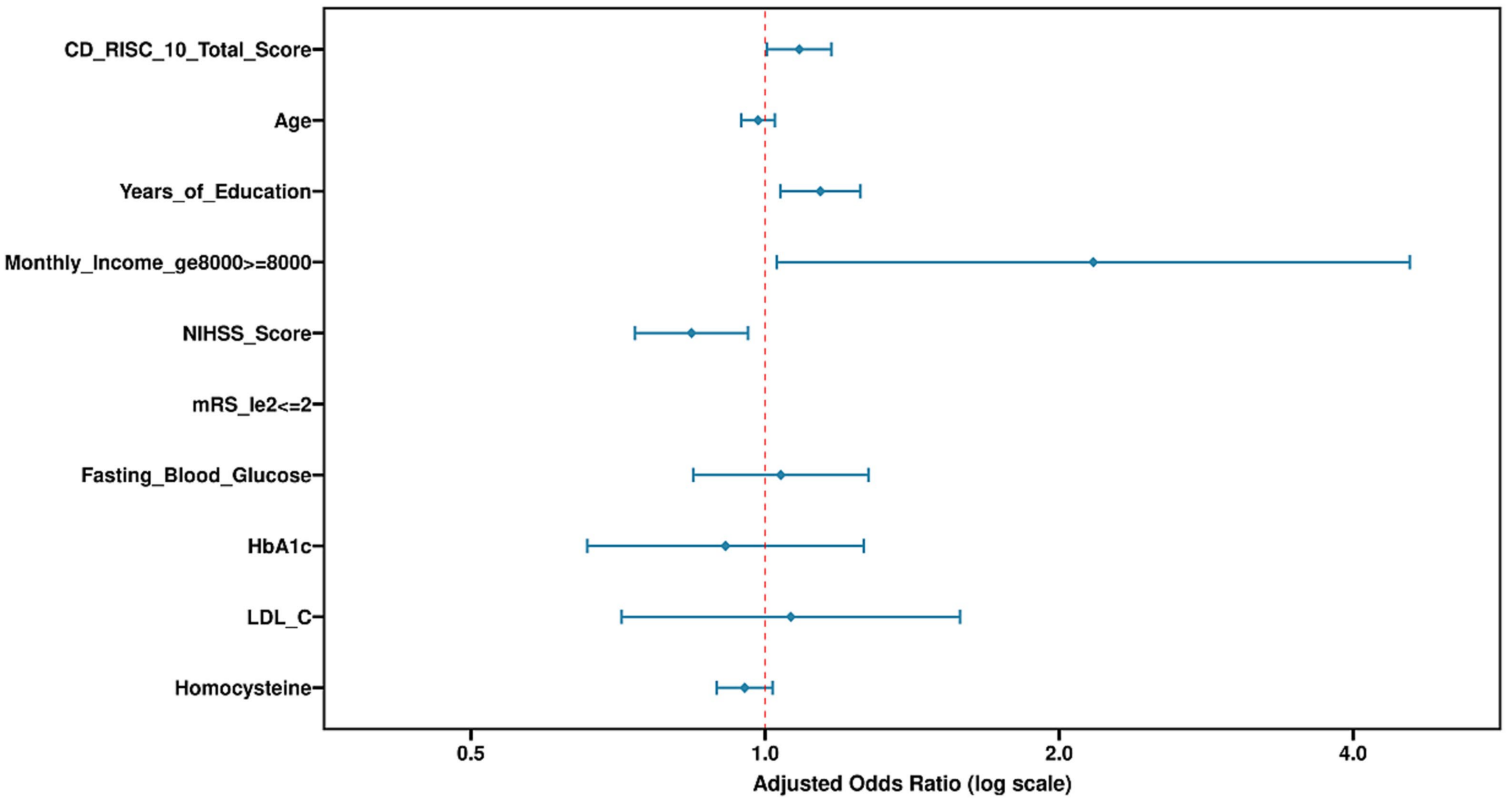
Multivariate logistic regression: factors associated with return to work.

Table 3 shows the multivariate logistic regression analysis, with the same dependent variable as in Table 2 and includes adjusted independent variables. The CD-RISC-10 total score, age, monthly income, and NIHSS score were identified as independent factors influencing Return to Work, with Wald *χ*^2^ values of 24.866, 8.543, 8.734, and 13.245, respectively (*p* < 0.05), and significant adjusted ORs (aOR). Years of education, mRS, fasting blood glucose, HbA1c, LDL-C, and homocysteine were not statistically significant. The constant term had no significant impact (see Table 3 and Figures 1–3).

**Table 3:** Multivariate logistic regression analysis of factors influencing return to work in young and middle-aged stroke survivors.

Variable	*β*-value	Std. error	Wald *χ*^2^ value	*p*-value	Adjusted OR (aOR)	95% CI
CD-RISC-10 total score (per 1-point increase)	0.139	0.028	24.866	<0.001	1.149	1.088–1.213
Age (per 1-year increase)	−0.052	0.018	8.543	0.003	0.949	0.917–0.982
Years of education (per 1-year increase)	0.071	0.042	2.842	0.092	1.073	0.989–1.165
Monthly income ≥8,000 CNY (vs. <8,000)	0.943	0.319	8.734	0.003	2.568	1.376–4.793
NIHSS score (per 1-point increase)	−0.201	0.055	13.245	<0.001	0.818	0.734–0.912
Baseline mRS ≤ 2 (vs. >2)	0.681	0.37	3.386	0.066	1.976	0.956–4.084
Fasting blood glucose (per 1 mmol/L increase)	−0.108	0.075	2.095	0.148	0.897	0.775–1.039
HbA1c (per 1% increase)	−0.185	0.115	2.579	0.108	0.831	0.663–1.041
LDL-C (per 1 mmol/L increase)	−0.213	0.152	1.96	0.162	0.808	0.600–1.088
Homocysteine (per 1 μmol/L increase)	−0.032	0.025	1.605	0.205	0.969	0.922–1.018
Constant	0.874	1.782	0.241	0.624	2.397	-

Sensitivity analyses using backward elimination and bootstrap resampling (1,000 replications) confirmed the robustness of the selected predictors, with the same set of variables retained in >95% of bootstrap samples.

#### 3.3.1 Model diagnostics and assumption checks

To ensure the validity of the multivariable logistic regression model, we conducted several diagnostic tests. Multicollinearity assessment revealed that all variance inflation factor (VIF) values were below 2.5, indicating no concerning collinearity among predictors. Specifically, the VIFs for NIHSS and mRS were 2.1 and 2.3, respectively, confirming that these two variables, despite their conceptual overlap, did not introduce substantial multicollinearity. The Box-Tidwell test for linearity of the logit showed that all continuous predictors (age, years of education, NIHSS, CD-RISC-10, fasting glucose, HbA1c, LDL-C, homocysteine) had non-significant interaction terms with their log-transformed versions (all *p* > 0.05), supporting the linearity assumption. These diagnostic checks confirm the appropriateness of the final logistic regression model presented in Table 3.

### 3.4 Stratified analysis of psychological resilience (CD-RISC-10) and return to work

Table 4 presents a stratified analysis based on psychological resilience (CD-RISC-10) levels, comparing Return to Work rates across different level groups. A significant trend was observed in RTW rates across the low, medium, and high-level groups (*p* for trend <0.001). The crude ORs for the medium and high-level groups were 4.889 and 6.967, respectively, indicating significantly higher RTW rates compared to the low-level group (see Table 4).

**Table 4:** Comparison of return to work rates across different psychological resilience level groups.

Psychological resilience level (CD-RISC-10)	Total (n)	RTW (n)	RTW rate (%)	Crude OR (95% CI)	*p*-value (trend)
Low level group (≤27 points)	84	24	28.6	1.00 (reference)	<0.001
Medium level group (28–33 points)	85	56	65.9	4.889 (2.566–9.316)	
High level group (≥34 points)	84	71	84.5	6.967 (3.508–13.835)	

### 3.5 Analysis of characteristics at different return to work time points

Table 5 compares the characteristics of the Early Return group (≤4 months) and the Late Return group (>4 months). The intergroup comparison before intervention showed that the Early Return group had a higher CD-RISC-10 total score (*t* = 5.468, *p* < 0.001), a lower baseline NIHSS score (*Z* = −3.952, *p* < 0.001), and a higher proportion of individuals with a monthly income ≥8,000 CNY (*χ*^2^ = 7.656, *p* = 0.006). Intragroup changes before and after intervention were not provided; the comparison is based solely on baseline characteristics (see Table 5).

**Table 5:** Comparison of characteristics of patients with different return to work time points.

Characteristic	Early return group (≤4 months, *n* = 73)	Late return group (>4 months, *n* = 58)	Statistical value	*p*-value
CD-RISC-10 total score, mean ± SD	34.82 ± 3.01	31.34 ± 4.22	*t* = 5.468	<0.001
Baseline NIHSS score, median (IQR)	3 (1, 5)	5 (3, 7)	*Z* = −3.952	<0.001
Monthly income ≥8,000 CNY, *n* (%)	58 (79.5%)	33 (56.9%)	*χ*^2^ = 7.656	0.006

### 3.6 Evaluation of the discriminative performance of prediction models

**Figure 4:**
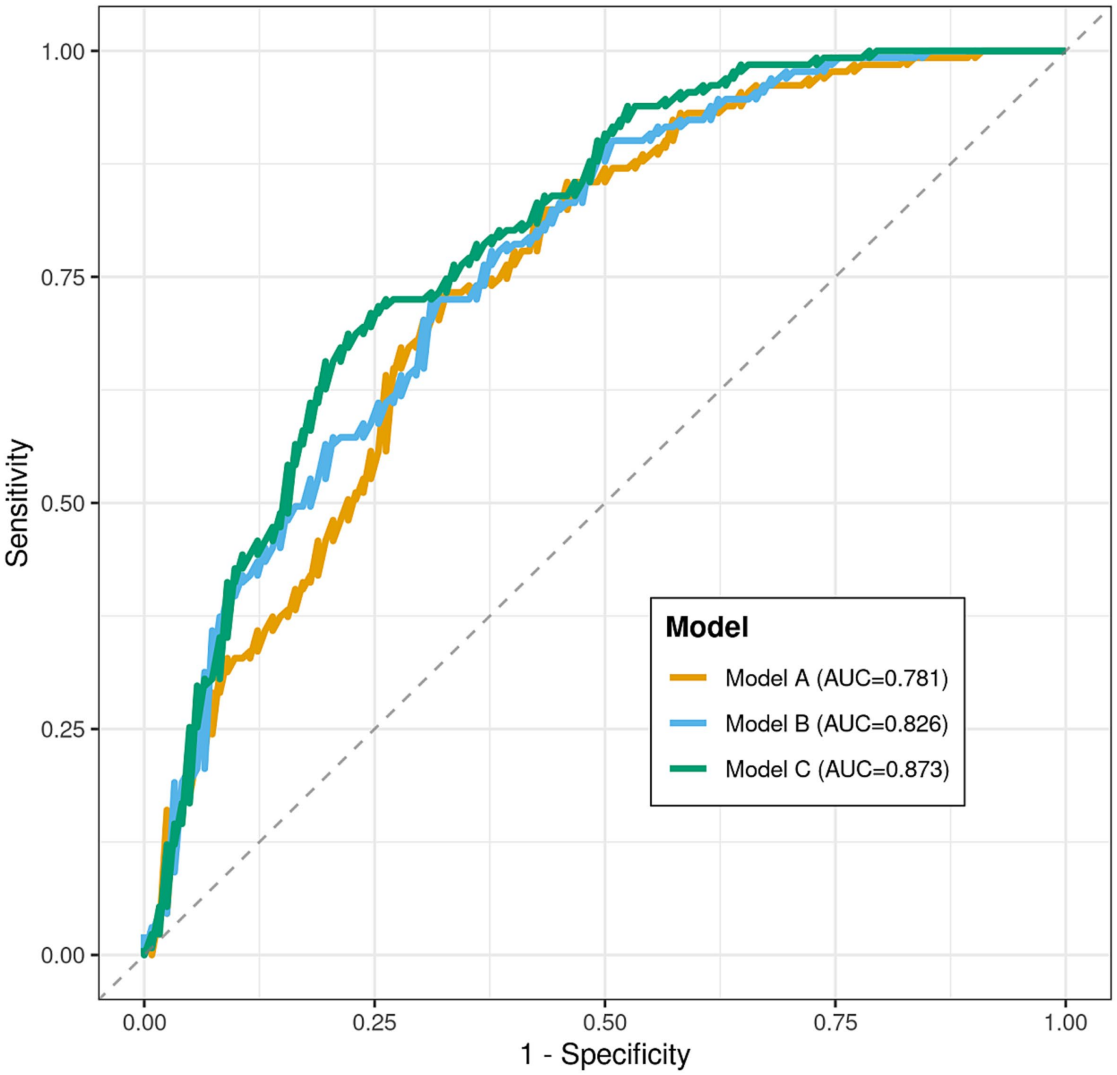
ROC curves for different prediction models of return to work.

Table 6 evaluates the discriminative performance of the prediction models for Return to Work status. Model C (including age, NIHSS score, monthly income, and CD-RISC-10 total score) achieved the highest AUC value of 0.873. Comparisons with Model A and Model B yielded *p* < 0.05, indicating that the comprehensive model (Model C) had the best predictive performance. The AUC values for Model A and Model B were 0.781 and 0.826, respectively (see Table 6 and Figure 4).

**Table 6:** Comparison of discriminative performance of different prediction models for return to work status (DeLong test for AUC comparisons).

Prediction model	Variables included	AUC value	Std. error	95% confidence interval	*p*-value (vs. Model C)
Model A: clinical model	Age, NIHSS score	0.781	0.029	0.724–0.838	<0.001
Model B: clinical-socioeconomic model	Age, NIHSS score, monthly income	0.826	0.026	0.775–0.877	0.013
Model C: comprehensive model	Age, NIHSS score, monthly income, CD-RISC-10 total score	0.873	0.022	0.830–0.916	Reference

*p*-values for AUC comparisons vs. Model C were calculated using the DeLong test for correlated ROC curves.

## 4 Discussion

This study aimed to investigate the multidimensional factors influencing Return to Work (RTW) in young and middle-aged stroke survivors, with a particular focus on the role of psychological resilience (16). The findings clearly demonstrate that RTW is a complex process constrained by demographic characteristics, clinical disease severity, socioeconomic status, and individual psychological resources. A core discovery is that a higher level of psychological resilience is a significant protective factor for RTW, and its predictive power is independent of traditional clinical and socioeconomic variables (17). Additionally, age, severity of post-stroke neurological deficits, and economic income were also identified as independent factors influencing RTW. Collectively, these findings highlight the multidimensional nature of factors influencing post-stroke RTW, encompassing demographic, clinical, and psychological domains. This underscores the necessity and urgency of moving beyond a purely biomedical approach and incorporating psychosocial factor screening and intervention into routine clinical rehabilitation assessment and management to better address the complex needs of young and middle-aged stroke survivors.

The comparison of baseline characteristics revealed that the group of survivors who did not return to work presented a more challenging risk profile. This group was older, had fewer years of education, lower economic income, more severe neurological deficits, and poorer control of physiological and metabolic indicators such as blood glucose and lipids (18). Notably, a significant disparity in psychological resilience levels existed between the two groups, with the non-RTW group exhibiting markedly lower resilience scores. These differences are not isolated; they likely intertwine to form a negative cycle hindering RTW. For instance, lower educational attainment and economic resources may limit access to high-quality rehabilitation services and information, while poorer physiological indicators reflect suboptimal overall health management. These factors, in turn, may interact with lower psychological resilience, undermining an individual’s intrinsic motivation and resilience to cope with post-stroke challenges (19).

Univariate analysis further confirmed significant associations between multiple variables and RTW status, spanning a broad spectrum from physiological to psychological domains. Increasing age, worsening neurological deficits, abnormal glucose and lipid metabolism, and elevated homocysteine levels were all negative predictors of RTW (20). Conversely, more years of education, higher economic income, and better functional status (mRS ≤ 2) constituted favorable conditions. Among all factors, psychological resilience demonstrated a notably strong association, with the change in the odds ratio per unit increase being particularly striking. This result suggests that psychological resilience may be not merely a concomitant variable but a core resource capable of buffering against the impact of various adverse factors. Previous research has also found that higher resilience is associated with better post-stroke adaptation and adherence to health behaviors, providing a potential behavioral explanatory pathway for the strong association observed in this study (21).

After controlling for numerous confounding factors, the multivariate regression model highlighted the strong, independent contribution of psychological resilience. Together with age, monthly income, and NIHSS score, it formed the core predictive model for RTW (22). This finding is significant because it moves beyond clinical intuition by quantifying the unique predictive power of a psychosocial factor. It indicates that even after accounting for disease severity (NIHSS) and basic socioeconomic status (income), an individual’s psychological resources, as measured by the CD-RISC-10, provide additional, meaningful information about their likelihood of returning to work. Several mechanisms may explain the observed association between psychological resilience and RTW. Individuals with higher resilience may employ more adaptive coping strategies, maintain greater engagement with rehabilitation, and navigate workplace reintegration challenges more effectively (23). However, as our observational design cannot establish causality, these proposed mechanisms remain speculative and require testing in mechanistic studies.

Crucially, unlike fixed factors such as age, psychological resilience is considered potentially modifiable (24). The identification of resilience as a strong, independent predictor of RTW generates the hypothesis that interventions targeting resilience might improve RTW outcomes. This hypothesis requires testing in randomized controlled trials before clinical recommendations can be made. If confirmed in interventional studies, evidence-based approaches shown to enhance resilience in other chronic disease populations—such as cognitive-behavioral therapy, mindfulness-based interventions, or strength-based counseling (25)—could potentially be adapted and integrated into multidisciplinary stroke rehabilitation programs. Such interventions, if proven effective, might equip patients with skills to better utilize available medical and social support, potentially enhancing the effectiveness of traditional rehabilitation (26). In contrast, some physiological and biochemical indicators that were significant in the univariate analysis did not enter the final model. This may imply that their influence is partly mediated through their effect on neurological function or through interaction with psychosocial factors, with their direct effect being overshadowed by more powerful predictive variables in the comprehensive model.

The stratified analysis presented the dose–response relationship between psychological resilience and RTW rate in a more intuitive manner. As resilience scores increased from low to high levels, the RTW rate showed a stepwise and significant rise, with the odds ratios for the medium and high-level groups increasing sharply compared to the low-level group (24). This dose–response relationship supports conceptualizing psychological resilience as a quantifiable, continuous protective factor rather than a simple dichotomous trait. It suggests the existence of clear threshold effects and windows of opportunity for clinical intervention: targeted enhancement interventions for survivors with low resilience may yield the greatest marginal benefit. Previous studies have found that interventions based on cognitive-behavioral therapy or mindfulness can effectively improve psychological resilience levels in patients with chronic diseases, providing a theoretical basis and feasibility reference for implementing similar interventions in the stroke population to promote RTW in the future (25).

The characteristic differences between the early and late return groups further refined the key elements influencing the RTW process. Early returners not only possessed higher psychological resilience but also had relatively milder neurological damage in the acute phase of stroke and more abundant economic resources (26). These three factors may constitute an “advantageous combination” that accelerates recovery: milder neurological damage provides a biological foundation for rapid functional recovery, sufficient economic resources ensure the continuity and quality of rehabilitation, and high resilience supplies the psychological engine to overcome early setbacks and actively plan for return. This finding implies that the timing of RTW is influenced by the synergistic effect of multiple factors. Identifying individuals with this “advantageous combination” of features can help clinicians early screen for those more likely to return to society quickly and provide them with more targeted, intensified rehabilitation pathways aimed at achieving RTW as soon as possible (27).

The comparison of prediction model performance provides empirical evidence for developing clinically practical RTW prediction tools. The stepwise and significant improvement in discriminative performance—from the basic model containing only clinical variables, to the model incorporating socioeconomic factors, and finally to the comprehensive model integrating psychological resilience—holds important implications (28). It quantitatively demonstrates that relying solely on disease severity indicators is insufficient for predicting RTW outcomes in young and middle-aged stroke patients. Including socioeconomic factors improves prediction, and introducing the variable of psychological resilience on this basis can lead to a substantial leap in predictive accuracy. The excellent discriminative performance of the comprehensive model indicates that an assessment framework integrating physiological, social, and psychological dimensions best aligns with the complex nature of the RTW outcome (29). This points the way for future development of multidimensional prediction scores or clinical decision support systems that include brief assessment tools for psychological resilience.

This study also has several limitations. First, its single-center, observational design can reveal associations between factors but cannot establish definitive causal relationships. Second, although our attrition rate was low (2.7%), the potential for bias from the seven participants lost to follow-up cannot be entirely excluded, despite the absence of significant baseline differences between them and the analyzed cohort. Second, the relatively homogeneous sample source may limit the generalizability of the findings to different healthcare systems and cultural contexts. Future research needs to validate the causal pathways between psychological resilience and RTW through multi-center, prospective longitudinal designs and explore its underlying neurophysiological and behavioral mechanisms. Furthermore, developing and validating simple, effective psychological resilience screening tools applicable in the early stages of stroke, as well as designing evidence-based, structured intervention programs aimed at enhancing the psychological resilience of young and middle-aged stroke survivors and testing their effect on improving RTW outcomes, will be highly valuable clinical research directions.

In summary, this study confirms that psychological resilience is a key and independent psychological protective factor influencing RTW in young and middle-aged stroke survivors. Return to Work is systematically influenced by multiple factors, including age, neurological deficits, economic status, and psychological resilience. Clinical practice should move beyond the traditional biomedical model toward a comprehensive rehabilitation framework that integrates psychosocial assessment. Early identification of individuals with insufficient psychological resilience, combined with routine neurological rehabilitation and vocational counseling to implement multidimensional, personalized intervention strategies, is of great significance for optimizing the long-term social functional prognosis of young and middle-aged stroke patients.

## Data Availability

The datasets presented in this study are not publicly available due to ethical and privacy restrictions. The data contain sensitive patient health information that could compromise participant privacy and confidentiality. Requests to access the datasets should be directed to corresponding author, and will be considered on a case-by-case basis subject to approval by the Medical Ethics Committee of Fuyang People’s Hospital. Requests to access the datasets should be directed to Xiaoxi Tan, 18756883009@163.com.
